# Supplementary material for: Traditional East Asian Herbal Medicine for Post-Stroke Insomnia: A Systematic Review and Meta-Analysis of Randomized Controlled Trials
Source: Int J Environ Res Public Health. 2022 Feb 3;19(3):1754. doi: 10.3390/ijerph19031754 (PMC8834856; doi:10.3390/ijerph19031754)
Supplement: Supplementary file 1 [file ijerph-19-01754-s001.zip › Figure S1.pdf]

(A) excluding studies with high ROB

Study or Subgroup	*herbal medicine* Mean	SD	Total	*psychotropic drug* Mean	SD	Total	Weight	Mean Difference IV, Random, 95% CI
Chen(2010)	6.1	2.23	42	7.5	2.94	40	8.3%	-1.40 [-2.53, -0.27]
Chen(2018)	5.64	0.66	30	8.58	0.95	30		Not estimable
Dai(2020)	7	5	30	8	2.25	30	6.1%	-1.00 [-2.96, 0.96]
Gao(2016)	8.25	2.54	20	14	2.29	20	7.3%	-5.75 [-7.25, -4.25]
Gao(2021)	4.26	1.08	40	6.63	1.27	40	9.7%	-2.37 [-2.89, -1.85]
Guo(2017)	6.3	2.78	30	6.77	2.77	30		Not estimable
Liu(2011)	4.91	4.732	30	7.77	5.1	30		Not estimable
Mei(2016)	5.66	0.67	70	6.83	0.84	70		Not estimable
Qin(2014)	8.86	3.12	30	13.13	2.45	30	7.5%	-4.27 [-5.69, -2.85]
Qiu(2019)	8.13	3.55	30	9.77	3.84	30	6.3%	-1.64 [-3.51, 0.23]
Wang(2019a)	7.83	2.41	30	8.1	1.83	30	8.4%	-0.27 [-1.35, 0.81]
Wang(2019b)	7.21	2.03	43	7.69	2.15	42	8.9%	-0.48 [-1.37, 0.41]
Xu(2011)	6.2	1.91	30	6.12	1.82	30		Not estimable
Xu(2012)	6.56	1.92	31	9.22	2.24	31		Not estimable
Xu(2019)	8.38	1.06	50	10.05	1.38	50	9.7%	-1.67 [-2.15, -1.19]
Yin(2017)	6.2	1.9	30	8.61	1.81	30	8.8%	-2.41 [-3.35, -1.47]
Zhang(2017a)	6.57	1.17	30	9.77	1.86	30	9.2%	-3.20 [-3.99, -2.41]
Zhao(2021)	7.32	1.83	40	9.54	1.76	40		Not estimable
Zhou(2012)	7.49	0.67	60	7.98	1.41	62	9.9%	-0.49 [-0.88, -0.10]
**Total (95% CI)**			**435**			**434**	**100.0%**	**-2.03 [-2.79, -1.27]**

Heterogeneity: Tau² = 1.49; Chi² = 113.93, df = 11 (P < 0.00001); I² = 90%
Test for overall effect: Z = 5.22 (P < 0.00001)

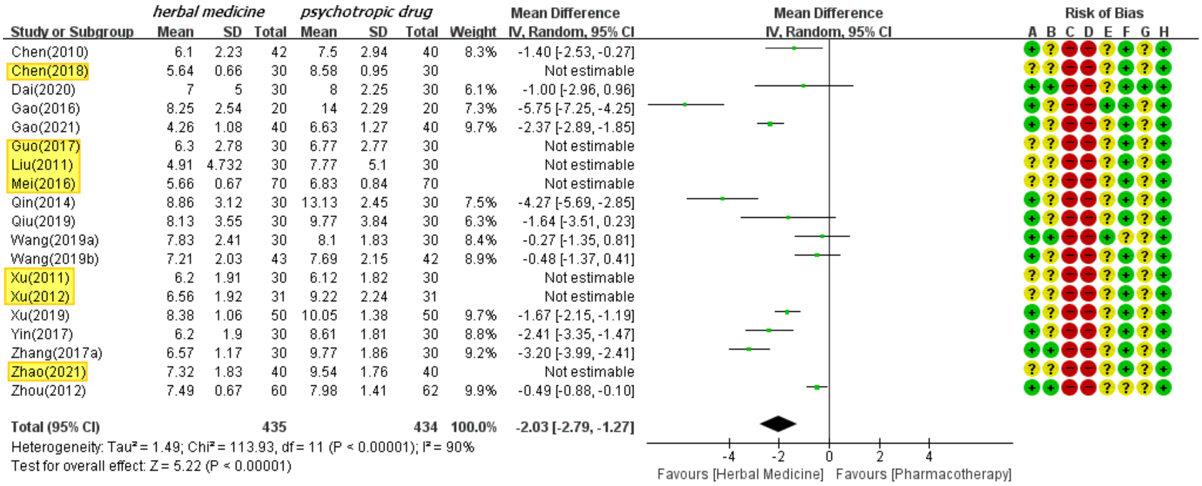

Risk of bias legend
(A) Random sequence generation (selection bias)
(B) Allocation concealment (selection bias)
(C) Blinding of participants
(D) Blinding of personnel
(E) Blinding of outcome assessment (detection bias)
(F) Incomplete outcome data (attrition bias)
(G) Selective reporting (reporting bias)
(H) Other bias

(B) excluding outlier that were numerically distant from the rest data

Study or Subgroup	*herbal medicine* Mean	SD	Total	*psychotropic drug* Mean	SD	Total	Weight	Mean Difference IV, Random, 95% CI
Chen(2010)	6.1	2.23	42	7.5	2.94	40	5.6%	-1.40 [-2.53, -0.27]
Chen(2018)	5.64	0.66	30	8.58	0.95	30	7.3%	-2.94 [-3.35, -2.53]
Dai(2020)	7	5	30	8	2.25	30	3.6%	-1.00 [-2.96, 0.96]
Gao(2016)	8.25	2.54	20	14	2.29	20	0.0%	-5.75 [-7.25, -4.25]
Gao(2021)	4.26	1.08	40	6.63	1.27	40	7.1%	-2.37 [-2.89, -1.85]
Guo(2017)	6.3	2.78	30	6.77	2.77	30	4.8%	-0.47 [-1.87, 0.93]
Liu(2011)	4.91	4.732	30	7.77	5.1	30	2.7%	-2.86 [-5.35, -0.37]
Mei(2016)	5.66	0.67	70	6.83	0.84	70	7.6%	-1.17 [-1.42, -0.92]
Qin(2014)	8.86	3.12	30	13.13	2.45	30	0.0%	-4.27 [-5.69, -2.85]
Qiu(2019)	8.13	3.55	30	9.77	3.84	30	3.7%	-1.64 [-3.51, 0.23]
Wang(2019a)	7.83	2.41	30	8.1	1.83	30	5.7%	-0.27 [-1.35, 0.81]
Wang(2019b)	7.21	2.03	43	7.69	2.15	42	6.2%	-0.48 [-1.37, 0.41]
Xu(2011)	6.2	1.91	30	6.12	1.82	30	6.1%	0.08 [-0.86, 1.02]
Xu(2012)	6.56	1.92	31	9.22	2.24	31	5.8%	-2.66 [-3.70, -1.62]
Xu(2019)	8.38	1.06	50	10.05	1.38	50	7.2%	-1.67 [-2.15, -1.19]
Yin(2017)	6.2	1.9	30	8.61	1.81	30	6.1%	-2.41 [-3.35, -1.47]
Zhang(2017a)	6.57	1.17	30	9.77	1.86	30	6.5%	-3.20 [-3.99, -2.41]
Zhao(2021)	7.32	1.83	40	9.54	1.76	40	6.5%	-2.22 [-3.01, -1.43]
Zhou(2012)	7.49	0.67	60	7.98	1.41	62	7.4%	-0.49 [-0.88, -0.10]
**Total (95% CI)**			**646**			**645**	**100.0%**	**-1.60 [-2.10, -1.10]**

Heterogeneity: Tau² = 0.86; Chi² = 143.79, df = 16 (P < 0.00001); I² = 89%
Test for overall effect: Z = 6.22 (P < 0.00001)

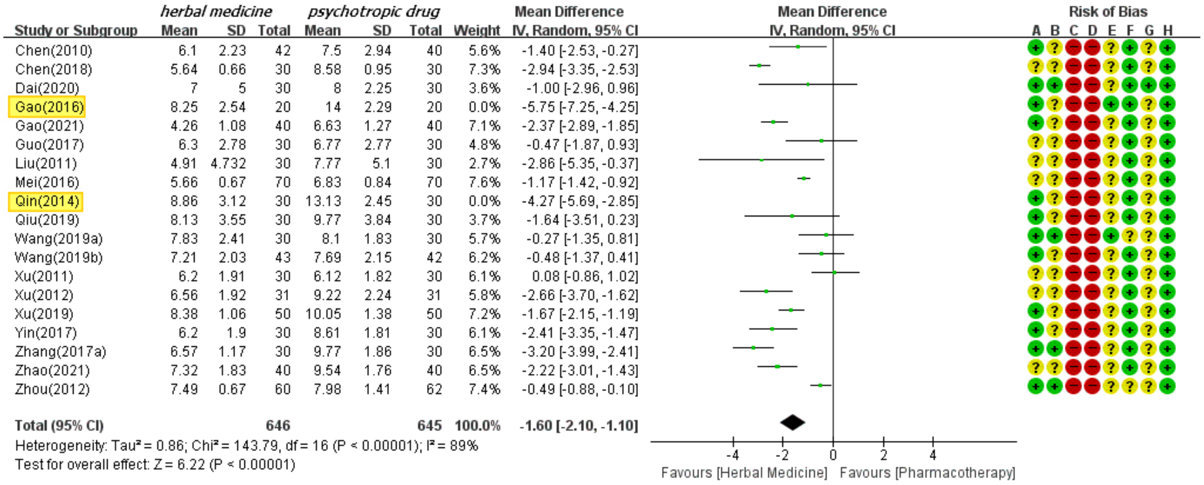

Risk of bias legend
(A) Random sequence generation (selection bias)
(B) Allocation concealment (selection bias)
(C) Blinding of participants
(D) Blinding of personnel
(E) Blinding of outcome assessment (detection bias)
(F) Incomplete outcome data (attrition bias)
(G) Selective reporting (reporting bias)
(H) Other bias

Figure S1. Forest plots for comparison of PSQI between herbal medicine and psychotropic drug groups. Sensitivity analysis after (A) excluding studies with high ROB and (B) excluding outlier that were numerically distant from the rest data
