# Supplementary material for: Traditional East Asian Herbal Medicine for Post-Stroke Insomnia: A Systematic Review and Meta-Analysis of Randomized Controlled Trials
Source: Int J Environ Res Public Health. 2022 Feb 3;19(3):1754. doi: 10.3390/ijerph19031754 (PMC8834856; doi:10.3390/ijerph19031754)
Supplement: Supplementary file 1 [file ijerph-19-01754-s001.zip › Figure S2.pdf]

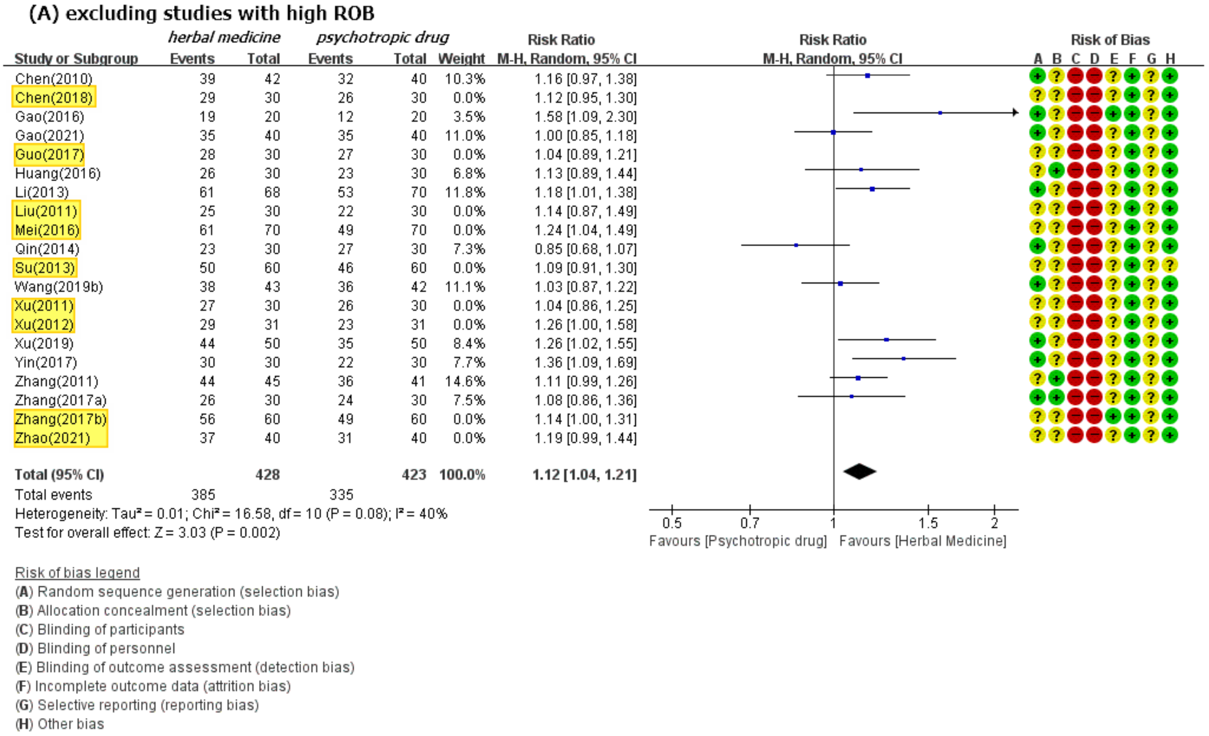

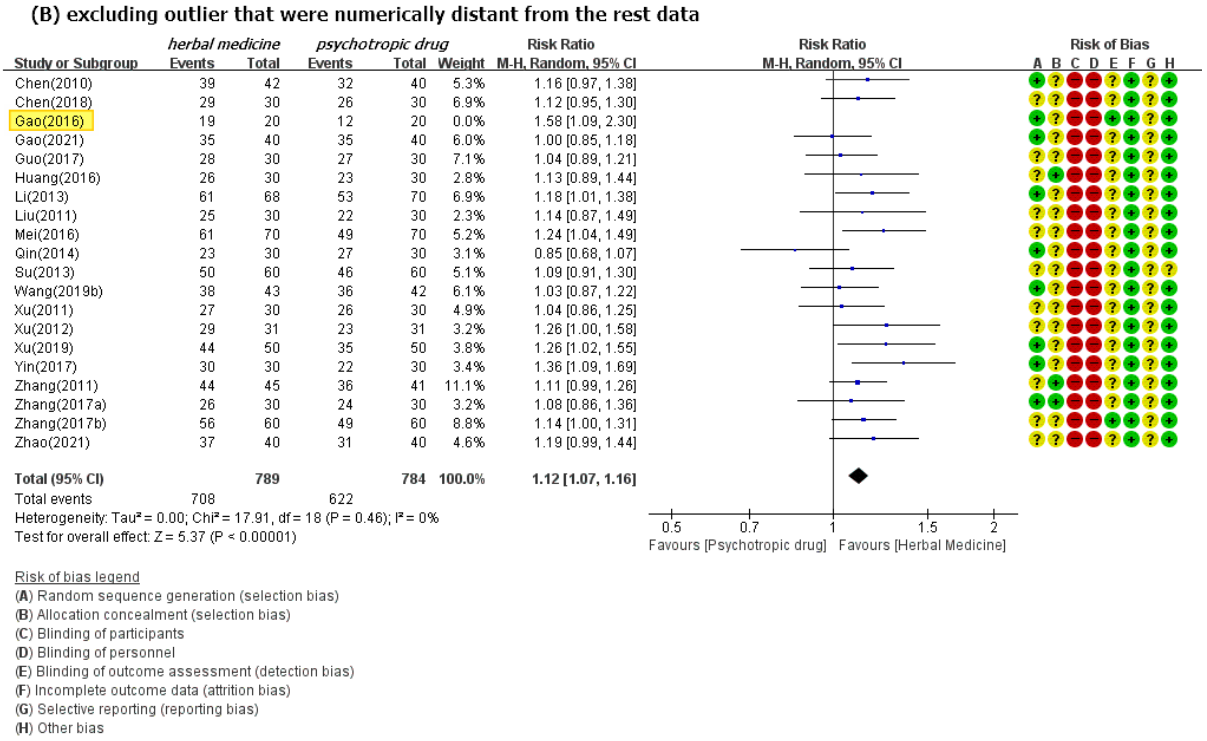

Figure S2. Forest plots for comparison of TER between herbal medicine and psychotropic drug groups. Sensitivity analysis after (A) excluding studies with high ROB and (B) excluding outlier that were numerically distant from the rest data
