# Supplementary material for: Traditional East Asian Herbal Medicine for Post-Stroke Insomnia: A Systematic Review and Meta-Analysis of Randomized Controlled Trials
Source: Int J Environ Res Public Health. 2022 Feb 3;19(3):1754. doi: 10.3390/ijerph19031754 (PMC8834856; doi:10.3390/ijerph19031754)
Supplement: Supplementary file 1 [file ijerph-19-01754-s001.zip › Figure S3.pdf]

	herbal medicine			*psychotropic drug*				Mean Difference
Study or Subgroup	Mean	SD	Total	Mean	SD	Total	Weight	IV, Fixed, 95% CI
Guo(2017)	6.82	2.58	30	7.73	2.98	30	9.6%	-0.91 [-2.32, 0.50]
Zhao(2021)	7.87	1.88	40	11.2	1.92	40	27.5%	-3.33 [-4.16, -2.50]
Zhou(2012)	4.5	1.5	60	7.8	1.6	62	62.9%	-3.30 [-3.85, -2.75]
**Total (95% CI)**			**130**			**132**	**100.0%**	**-3.08 [-3.52, -2.64]**

Heterogeneity: Chi² = 10.05, df = 2 (P = 0.007); I² = 80%
Test for overall effect: Z = 13.83 (P < 0.00001)

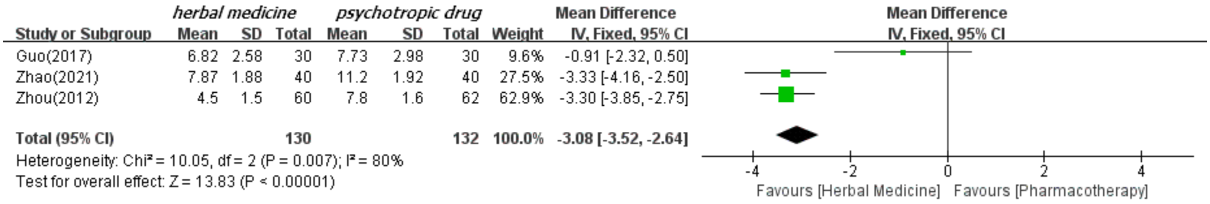

Figure S3. Forest plots for comparison of PSQI between herbal medicine and psychotropic drug groups in the follow-up studies.
