# Supplementary material for: Traditional East Asian Herbal Medicine for Post-Stroke Insomnia: A Systematic Review and Meta-Analysis of Randomized Controlled Trials
Source: Int J Environ Res Public Health. 2022 Feb 3;19(3):1754. doi: 10.3390/ijerph19031754 (PMC8834856; doi:10.3390/ijerph19031754)
Supplement: Supplementary file 1 [file ijerph-19-01754-s001.zip › Figure S4.pdf]

	herbal medicine		psychotropic drug			Risk Ratio
**Study or Subgroup**	**Events**	**Total**	**Events**	**Total**	**Weight**	**M-H, Random, 95% CI**
Chen(2010)	3	42	8	40	34.1%	0.36 [0.10, 1.25]
Dai(2020)	1	30	6	30	12.7%	0.17 [0.02, 1.30]
Guo(2017)	1	30	3	30	0.0%	0.33 [0.04, 3.03]
Huang(2016)	2	30	7	30	24.2%	0.29 [0.06, 1.26]
Liu(2011)	0	30	6	30	0.0%	0.08 [0.00, 1.31]
Qiu(2019)	0	30	0	30		Not estimable
Wang(2019a)	1	30	6	30	12.7%	0.17 [0.02, 1.30]
Wang(2019b)	0	44	1	44	5.3%	0.33 [0.01, 7.97]
Xu(2012)	0	31	5	31	0.0%	0.09 [0.01, 1.58]
Zhang(2017a)	1	30	3	30	11.0%	0.33 [0.04, 3.03]
Zhao(2021)	0	40	0	40		Not estimable
**Total (95% CI)**		**236**		**234**	**100.0%**	**0.28 [0.13, 0.57]**
Total events	8		31			

Heterogeneity: Tau² = 0.00; Chi² = 0.68, df = 5 (P = 0.98); I² = 0%

Test for overall effect: Z = 3.45 (P = 0.0006)

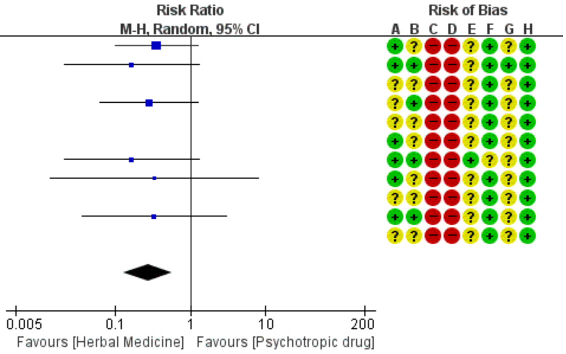

Risk of bias legend
(A) Random sequence generation (selection bias)
(B) Allocation concealment (selection bias)
(C) Blinding of participants
(D) Blinding of personnel
(E) Blinding of outcome assessment (detection bias)
(F) Incomplete outcome data (attrition bias)
(G) Selective reporting (reporting bias)
(H) Other bias

Figure S4. Forest plots for comparison of AE between herbal medicine and psychotropic drug groups. Sensitivity analysis after excluding studies with high ROB.
