# Supplementary material for: Traditional East Asian Herbal Medicine for Post-Stroke Insomnia: A Systematic Review and Meta-Analysis of Randomized Controlled Trials
Source: Int J Environ Res Public Health. 2022 Feb 3;19(3):1754. doi: 10.3390/ijerph19031754 (PMC8834856; doi:10.3390/ijerph19031754)
Supplement: Supplementary file 1 [file ijerph-19-01754-s001.zip › Table S2.pdf]

Table S2. Search terms used in each database

Medline via PubMed

	Searches	Results
#1	″Sleep″[MH] OR ″sleep wake disorders″[MH] OR sleep* OR insomnia* OR wakeful* OR sleepless* OR dyssomn*	273824
#2	"stroke"[MH] OR stroke	375208
#3	″Plants, Medicinal″[MH] OR ″Drugs, Chinese Herbal″[MH] OR ″Medicine, Chinese Traditional″[MH] OR ″Medicine, Kampo″[MH] OR ″Medicine, Korean Traditional″[MH] OR ″Herbal Medicine″[MH] OR ″Prescription Drugs″[MH] OR ″traditional Korean medicine″ OR ″traditional Chinese medicine″ OR ″Traditional oriental medicine″ OR ″Kampo medicine″ OR ″alternative medicine″ OR ″complementary medicine″ OR herb* OR decoction* OR botanic*	364699
#4	#1 AND #2 AND #3	**84**

EMBASE via Elsevier

	Searches	Results
#1	‘Sleep’/exp OR ‘sleep’ OR ‘sleep disorder’/exp OR ‘sleep disorder’ OR ‘insomnia*’ OR ‘wakeful*’ OR ‘sleepless*’ OR ‘dyssomn*’	496155
#2	'cerebrovascular disease'/exp OR 'cerebrovascular disease'	791840
#3	'stroke patient'/exp OR 'stroke patient'	37590
#4	‘Stroke’	493085
#5	‘medicinal plant’/exp OR ‘medicinal plant’ OR ‘herbaceous agent’/exp OR ‘herbaceous agent’ OR ‘chinese medicine’/exp OR ‘chinese medicine’ OR ‘kampo medicine’/exp OR ‘kampo medicine’ OR ‘kampo medicine (drug)’/exp OR ‘kampo medicine (drug)’ OR ‘korean medicine’/exp OR ‘korean medicine’ OR ‘herbal medicine’/exp OR ‘herbal medicine’ OR ‘prescription drug’/exp OR ‘prescription drug’ OR ‘oriental medicine’/exp OR ‘oriental medicine’ OR ‘alternative medicine’/exp OR ‘alternative medicine’ OR ‘complementary medicine’ OR ‘herb’/exp OR ‘herb’ OR ‘decoction*’ OR ‘botanic*’	614933
#7	#1 AND (#2 OR #3 OR #4) AND #5	**646**

CENTRAL

	Searches	Results
#1	MeSH descriptor: [Sleep] explode all trees	5900
#2	MeSH descriptor: [Sleep wake disorders] explode all trees	8521
#3	sleep* OR insomnia* OR wakeful* OR sleepless* OR dyssomn*	51134
#4	#1 OR #2 OR #3	51417
#5	MeSH descriptor: [Stroke] explode all trees	10417
#6	(stroke):ti,ab,kw	57872
#7	#5 OR #6	58304
#8	(#1 OR #2 OR #3) AND (#5 OR #6)	1050
#9	MeSH descriptor: [Plants, Medicinal] explode all trees	946
#10	MeSH descriptor: [Drugs, Chinese Herbal] explode all trees	3665
#11	MeSH descriptor: [Medicine, Chinese Traditional] explode all trees	1222
#12	MeSH descriptor: [Medicine, Kampo] explode all trees	46
#13	MeSH descriptor: [Medicine, Korean Traditional] explode all trees	33
#14	MeSH descriptor: [Herbal Medicine] explode all trees	63
#15	MeSH descriptor: [Prescription Drugs] explode all trees	108
#16	traditional Korean medicine OR traditional Chinese medicine OR Traditional oriental medicine OR Kampo medicine OR alternative medicine OR complementary medicine OR	51216

	herb* OR decoction* OR botanic*	
#17	#9 OR #10 OR #11 OR #12 OR #13 OR #14 OR #15 OR #16	52098
#18	#8 AND #17	**79**

AMED via EBSCO

	Searches	Results
#1	Sleep[SU] OR sleep wake disorders[SU] OR sleep*[TX] OR insomnia*[TX] OR wakeful*[TX] OR sleepless*[TX] OR dyssomn*[TX]	3
#2	stroke[SU]	37
#3	stroke[TX]	2
#4	Plants, Medicinal[SU] OR Drugs, Chinese Herbal[SU] OR Medicine, Chinese Traditional[SU] OR Medicine, Kampo[SU] OR Medicine, Korean Traditional[SU] OR Herbal Medicine[SU] OR Prescription Drugs[SU]	478
#5	traditional Korean medicine[TX] OR traditional Chinese medicine[TX] OR Traditional oriental medicine[TX] OR Kampo medicine[TX] OR alternative medicine[TX] OR complementary medicine[TX] OR herb*[TX] OR decoction*[TX] OR botanic*[TX]	26
#6	#1 AND (#2 OR #3) AND (#4 OR #5)	**22**

CINAHL via EBSCO

	Searches	Results
#1	Sleep[SU] OR sleep wake disorders[SU] OR sleep*[TX] OR insomnia*[TX] OR wakeful*[TX] OR sleepless*[TX] OR dyssomn*[TX]	42
#2	stroke[SU]	672
#3	stroke[TX]	72
#4	Plants, Medicinal[SU] OR Drugs, Chinese Herbal[SU] OR Medicine, Chinese Traditional[SU] OR Medicine, Kampo[SU] OR Medicine, Korean Traditional[SU] OR Herbal Medicine[SU] OR Prescription Drugs[SU]	363
#5	traditional Korean medicine[TX] OR traditional Chinese medicine[TX] OR Traditional oriental medicine[TX] OR Kampo medicine[TX] OR alternative medicine[TX] OR complementary medicine[TX] OR herb*[TX] OR decoction*[TX] OR botanic*[TX]	38
#6	#1 AND (#2 OR #3) AND (#4 OR #5)	**334**

PsycARTICLES via ProQuest

	Searches	Results
#1	Exact("sleep wake disorders")	228
#2	Exact("sleep")	935
#3	SU(Sleep) OR SU(sleep wake disorders) OR 'sleep*' OR 'insomnia*' OR 'wakeful*' OR 'sleepless*' OR 'dyssomn*'	17586
#4	Exact("stroke")	124
#5	Stroke	6261
#6	Exact("drugs, chinese herbal") OR "plants, medicinal" OR "prescription drugs" OR "medicine, chinese traditional" OR "alternative medicine" OR "medicinal herbs and plants"	155
#7	'traditional Korean medicine' OR 'traditional Chinese medicine' OR 'Traditional oriental medicine' OR 'Kampo medicine' OR 'alternative medicine' OR 'complementary medicine' OR 'herb*' OR 'decoction*' OR 'botanic*'	31075
#7	(#1 OR #2 OR #3) AND (#4 OR #5) AND (#6 OR #7)	**621**

OASIS

	Searches	Results
#1	(뇌졸중 OR 중풍) AND (불면 OR 수면) AND 한약	**2**

KISS

	Searches	Results
#1	(뇌졸중 OR 중풍) AND (불면 OR 수면) AND 한약	**2**

RISS

	Searches	Results
#1	(뇌졸중 OR 중풍) AND (불면 OR 수면) AND 한약	**1**

KMbase

	Searches	Results
#1	(뇌졸중 OR 중풍) AND (불면 OR 수면) AND 한약	**1**

KCI

	Searches	Results
#1	(뇌졸중 OR 중풍) AND (불면 OR 수면) AND 한약	**0**

CNKI

	Searches	Results
#1	(SU='中风'+'脑卒中') AND (SU='失眠'+'不寐'+'不眠'+'不睡'+'不得眠'+'不得卧') AND (SU='中药'+'中医药'+'本草'+'汤'+'丸'+'散'+'方'+'颗粒'+'胶囊'+'自拟')	**381**

Wanfang data

	Searches	Results
#1	(中风 OR 脑卒中) AND (失眠 OR 不寐 OR 不眠 OR 不睡 OR 不得眠 OR 不得卧) AND (中药 OR 中医药 OR 本草 OR 汤 OR 丸 OR 散 OR 方 OR 颗粒 OR 胶囊 OR 自拟)	**2297**

VIP

	Searches	Results
#1	M=(中风 OR 脑卒中) AND M=(失眠 OR 不寐 OR 不眠 OR 不睡 OR 不得眠 OR 不得卧) AND M=(中药 OR 中医药 OR 本草 OR 汤 OR 丸 OR 散 OR 方 OR 颗粒 OR 胶囊 OR 自拟)	**131**

	* M=题名或关键词	

CiNii

	Searches	Results
#1	(stroke OR "stroke" OR 中風 OR 脳卒中) AND (Sleep OR insomnia* OR wakeful* OR sleepless* OR dyssomn* OR 不眠 OR ふみん OR インソムニア OR ねぶそく) AND ("traditional Korean medicine" OR "traditional Chinese medicine" OR "Traditional oriental medicine" OR "Kampo medicine" OR "alternative medicine" OR "complementary medicine" OR herb* OR decoction* OR botanic* OR 漢方薬 OR ハーブ OR 散 OR 汤 OR 丸)	**8**
