# Supplementary material for: Traditional East Asian Herbal Medicine for Post-Stroke Insomnia: A Systematic Review and Meta-Analysis of Randomized Controlled Trials
Source: Int J Environ Res Public Health. 2022 Feb 3;19(3):1754. doi: 10.3390/ijerph19031754 (PMC8834856; doi:10.3390/ijerph19031754)
Supplement: Supplementary file 1 [file ijerph-19-01754-s001.zip › Table S3.pdf]

Table S3. Excluded studies after full-text review

1) not RCT: 14

1. 房妮妮. 百乐眠胶囊治疗脑卒中急性期失眠患者的临床疗效. 神经疾病与精神卫生. 2016(002):149-52.(retropsective chart review)
2. 张小健, 刘晶晶, 王迎昌, 焦雪蕾. 黄连阿胶汤辨证加减对阴虚火旺型老年卒中后失眠患者总体睡眠状态及失眠药物剂量的影响. 空军医学杂志. 2017;33(2):110-2.(retropsective chart review)
3. 符世纯. 大定风珠治疗中风后遗不寐 36 例. 中华中医药学刊. 1993(5):43.(before-after study)
4. 郭二霞. 镇肝熄风汤加减治疗中风后睡眠颠倒 36 例. 安徽中医临床杂志. 2002;14(6):456-7.(before-after study)
5. 刘耀东, 王敬华, 孙丽萍, 段海平, 姬广伟. 归脾汤加减治疗中风后失眠. 中国民间疗法. 2009;17(4):34.(case series)
6. 杨明胜. 自拟豁痰通瘀泻火汤治疗中风后失眠症 36 例. 中医杂志. 2010(S2):191.(case series)
7. 李东晓, 曹晓岚. 浅析温胆汤加减治疗中风后失眠. 健康必读（中旬刊）. 2011(11):63.(case series)
8. 郭铁. 中医药治疗中风后失眠. 中国中医急症. 2011;20(12).(case series)
9. 曾令烽, 邹元平, 黄小桃, 宓穗卿, 孔令朔, 王奇, et al. 中医药干预对改善脑卒中后睡眠障碍疗效与安全性的系统评价. 中华中医药杂志. 2015;30(05):1488-98.(systemtic review)
10. 王岳青. 从清热化痰法论治急性脑梗死后睡眠障碍的临床研究 [硕士]: 北京中医药大学; 2019.
11. 王红胜, 吴迪, 朱芳梅, 郝坤, 王建彬. 柴胡加龙骨牡蛎汤加减改善中风后失眠30例临床观察. 中国保健营养. 2019;29(29):24.(before-after study)
12. 武润梅. 陈改花运用自拟调和肝脾方治疗脑卒中后不寐. 中国民间疗法. 2019;27(14).(case series)
13. 张霜梅. 卒中后失眠的临床研究及其中医药治疗的Meta分析. 博士, 广州中医药大学, 2020.(systemtic review)
14. 胡孔翠, 胡谢. 酸枣仁汤加减方治疗脑卒中后失眠的效果观察. 当代医药论丛. 2019;17(8):200-1.(quasi-RCT)

2) not about PSI: 2

1. 王迎昌, 张小健, 刘银波. 疏肝健脾方治疗失眠症60例观察. 实用中医药杂志. 2013;29(12):991-2.
2. 华玉凡, 谭子虎. 六味地黄丸合交泰丸加味治疗不寐疗效观察. 湖北中医杂志. 2014(10):38-9.

3) accompany psychological problem other than insomnia: 7

1. 黄学娣; 蒋司晨; 樊萍; 胡小玲; 严毅; 程富香; 唐莉华. 柔肝安神汤治疗脑卒中后睡眠障碍临床观察. 光明中医 2020, 35, 2619-2621.(with moderate or higher depression(HAMD) and anxiety(HAMA))
2. 付燕琼. 柴胡加龙骨牡蛎汤治疗脑卒中后失眠的临床观察. 中国民间疗法 2020, 28, 46-48.(with moderate or higher depression(HAMD) and anxiety(HAMA))
3. 李雪. 柴胡加龙骨牡蛎汤对脑卒中后失眠患者睡眠质量及血清5-HT、DA的影响. 现代医学与健

康研究 2020, 4.(with moderate or higher depression(SDS) and anxiety(SAS))

4. Li HC, Chen XG, Tian X. [Analysis on somnipathy related factors in elderly patients with stroke and comparative study on the efficacy of treatment by traditional Chinese medicine and by estazolam]. Zhongguo Zhong Xi Yi Jie He Za Zhi. 2009;29(3):204-7.(with moderate or higher depression(HAMD) and anxiety(HAMA))

5. 汤瑞珠. 柴胡加龙骨牡蛎汤治疗中风后失眠随机平行对照研究. 实用中医内科杂志. 2017;31(4).(with moderate or higher depression(HAMD) and anxiety(HAMA))

6. Wang XCL, J., Xia HT, Zhai YZ, Wu XH. Clinical study of Huanglian-Wendan Decoction combined with Estazolam tablets in the treatment of post-stroke sleep disorders with syndrome of internal disturbance of phlegm-heat. International Journal of Traditional Chinese Medicine. 2020(04):319-20-21-22-23.(with moderate or higher depression(HAMD) and anxiety(HAMA))

7. 眭淑彦; 朱明锦; 张震中. 加味酸枣仁汤治疗卒中后认知障碍伴失眠对患者HPA轴功能、睡眠质量及认知功能的影响. 中国现代医生 2020, 58, 14-17+ 21.(with cognitive disorder)

4) not report diagnostic criteria of stroke: 5

1. 韩永强. 天王补心丸治疗脑卒中后失眠症60例疗效观察. 中国实用医药. 2011;6(21).

2. 杨金亮. 心脑宁胶囊治疗脑卒中后失眠的临床疗效观察. 北京医学. 2015;37(1).

3. 左艳丽. 《 回回药方 》 失荅刺知丸治疗中风后失眠40例临床观察. 中国民族医药杂志. 2015;21(7).

4. 郑聪. 祛痰安神汤治疗脑卒中后失眠的疗效观察. 内蒙古中医药. 2016;35(4).

5. 雷艳. 中西药结合治疗脑卒中后失眠患者的疗效. 医疗装备. 2016;29(12):144-,5.

5) not report diagnostic criteria of insomnia: 28

1. 刘健红. 自拟滋阴安神汤治疗脑卒中后失眠的临床观察. 四川中医. 2011;29(7):81-2.

2. 张小健, 刘晶晶, 王迎昌, 焦雪蕾, 周琦. 脑心同治法对老年卒中后失眠患者总体睡眠状态及失眠药物剂量的影响. 世界中医药. 2017(2):269-71.

3. 黄宇. 百乐眠胶囊治疗老年脑卒中后失眠的效果. 神经疾病与精神卫生. 2017 May 18;17(3).

4. 黄伟, 滕海英, 毛媛媛. 疏肝解郁胶囊联合艾司唑仑治疗卒中后睡眠障碍的疗效观察. 中西医结合心脑血管病杂志. 2018;8.

5. 冯蓓蕾, 王翘楚, 李正元. 落花安神合剂治疗脑卒中后失眠症的临床观察. 江苏中医药. 2004;25(7):21-2.

6. 吴济建, 俞亮, 金淑玲. 中风后不寐的中西医治疗. 浙江中西医结合杂志. 2006;16(8):520-..

7. 韩玉林. 脑卒中患者失眠的中西医结合治疗. 吉林医学. 2008;29(23):2242-3.

8. 王震华, 黄合, 金兰英. 稳心颗粒治疗脑卒中后睡眠障碍 66 例临床观察. 中国民族民间医药杂志. 2010(2):96-8.

9. 黄李双, 老膺荣, 黄红. 辨证调护治疗脑卒中后睡眠障碍 139 例疗效观察. 新中医. 2010(8):89.

10. 关运祥. 酸枣仁汤加减治疗脑卒中后失眠30例. 中医临床研究. 2012(19):97-8.

11. 王乃梅. 帕罗西汀联合百乐眠治疗脑卒中睡眠障碍的临床观察. 中国医药导刊. 2013(6):992-,4.

12. 李红. 金匮肾气丸联合艾司唑仑治疗脑卒中后失眠随机平行对照研究. 实用中医内科杂志. 2014(6).

13.	郑喜英, 姚淑文, 张群威, 程志忠. 参芪五味子片联合黛力新对脑卒中后失眠的影响. 中国实用神经疾病杂志. 2015(6):122-3.

14.	吕昕. 舒肝解郁胶囊联合右佐匹克隆治疗卒中后睡眠障碍的临床研究. 中西医结合心脑血管病杂志. 2016;14(13).

15.	郑晶. 乌灵胶囊联合草酸艾司西酞普兰治疗卒中后睡眠障碍的临床研究 [硕士]: 湖北中医药大学; 2017.

16.	姚财文. 落花生枝叶制剂治疗中风后失眠的临床疗效观察. 内蒙古中医药. 2018;37(1):24-5.

17.	郑萍红. 血府安神汤治疗中风后遗症期痰瘀互结型不寐的临床疗效观察. 中国全科医学. 2018;0(S1).

18.	李悦. 酸枣仁汤联合早期康复护理对脑出血睡眠及质量的影响. 中国中医药现代远程教育. 2019;17(15):114-6.

19.	付晓波. 柴胡加龙骨牡蛎汤治疗脑卒中后失眠患者的临床疗效. 医疗装备. 2019;32(20):115-6.

20.	魏雪琴. 参芪五味子胶囊联合阿普唑仑治疗脑卒中后合并失眠多梦的临床研究. 世界睡眠医学杂志. 2019;6(2).

21.	莫柏威. 柴胡加龙骨牡蛎汤治疗中风后失眠的效果观察. 内蒙古中医药. 2020;39(3).

22.	任敬丽. 血府安神汤治疗中风后遗症期痰瘀互结型不寐的临床疗效观察. 健康大视野. 2020;4.

23.	周美红. 中药干预改善脑卒中后睡眠障碍临床观察. 新中医. 2016;48(9):16-7.

24.	周媛. 养心安神汤治疗脑卒中后失眠临床观察. 辽宁中医药大学学报. 2015;17(8).

25.	易世宏. 安眠方对脑卒中恢复期失眠和神经功能康复的影响. 中国康复医学杂志. 2008;23(5).

26.	莫柏威. 柴胡加龙骨牡蛎汤治疗中风后失眠的效果观察. 内蒙古中医药 2020, 39, 59-60.

27.	孙巧杰. 酸枣仁汤加减治疗中风后失眠的临床研究. 中医临床研究. 2018;10(6):57-8.

28.	张爱萍. 活血安神方治疗缺血性脑卒中后失眠的临床观察. *中医临床研究*, 2016, 8.28: 63-64.

6) comparison of different HMs: 3

1.	刘宏雅, 薛辉. 天智颗粒治疗脑卒中后失眠症的疗效观察. 中国实用神经疾病杂志. 2008;11(10):117-8.

2.	曹泽慧. 分析比较六味地黄丸和奥氮平对老年痴呆精神行为 症状的改善作用. *医药前沿*. 2018;8(4):320.

3.	王敏. 加味菖蒲郁金汤治疗脑卒中后睡眠障碍（痰瘀阻窍证）的临床观察 [硕士]: 湖北中医药大学; 2016.

7) using herbal injection as routine care for stoke: 1

1 .	郑丽. 柴胡加龙骨牡蛎汤加味治疗缺血性中风后失眠36例临床观察. 中医临床研究. 2015(21):47-8.(combined with herbal injection)

8) Combined therapy with HM and psychotropic drugs: 13

1.	崔译心, 王京军, 阎晓悦. 补心养肝方结合西药治疗脑梗塞后失眠的临床观察. 内蒙古中医药. 2017;36(17):13.

2.	乌灵胶囊联合右佐匹克隆治疗脑卒中后失眠疗效观察. 中国药业 2021, 30.

3. 牛雁. 中西医结合治疗脑卒中后失眠临床观察. 光明中医. 2018;18.

4. 赵欣,姬孟艳,董强.加味丹栀逍遥散对中风后失眠患者睡眠质量及生活质量影响[J].检验医学与临床,2020,17(11):1535-1537.

5. 朱作权, 桂心, 杨叔英, 刘露. 养血清脑颗粒联合右佐匹隆片治疗急性脑卒中后失眠疗效观察. 中国药业. 2018;11.

6. 周城林,刘琼,梅琰,陈毅,龙再慧.归脾汤加味联合小剂量曲唑酮治疗脑卒中后失眠及对血脂及血流动力学的影响[J].中医临床研究,2020,12(21):53-56.

7. 高振忠, 宋立公. 柴胡龙牡胶囊治疗急性中风后失眠. 中医临床研究. 2010;2(23):91-2.

8. 周荣. 天王补心汤治疗缺血性脑卒中后失眠（阴虚火旺型）的临床疗效评价 [硕士]: 浙江中医药大学; 2015.

9. 刘立斌. 乌灵胶囊联合右佐匹克隆治疗脑卒中后失眠疗效观察. 中国药业 2021, 30.

10. 付俊丽; 封臻; 张双勇. 疏肝汤结合黛力新治疗老年缺血性卒中后抑郁临床观察. 光明中医 2021, 9.

11. 赵欣; 姬孟艳; 董强. 加味丹栀逍遥散对中风后失眠患者睡眠质量及生活质量影响. 检验医学与临床 2020, 17, 1535-1537.

12. 权东海. 柴胡加龙骨牡蛎汤治疗中风后失眠的效果评价. 东方药膳 2020, 22.

13. 周城林. 归脾汤加味联合小剂量曲唑酮治疗脑卒中后失眠及对血脂及血流动力学的影响. 中医临床研究 2020, 12.

9) using other intervention other than HM, psychotropic drugs, and routine care for stoke: 5

1. 王建军, 孙玫. 中西医结合治疗缺血性脑卒中失眠症疗效观察. 实用中医药杂志. 2017;33(6):700-1.(combined with psychotherapy)

2. 谭艳榕, 江浩. 老年脑卒中失眠患者的中药治疗及护理对策. 山东医药. 2003;43(23):57.(with rehabilitation not routine care for stoke)

3. 高惠艳. 辨证施护对缺血性中风后失眠的疗效观察. 光明中医. 2016;31(20):3016-8.(combined with massage, counselling, diet, lifestyle modification)

4. 王晓娟. 失眠认知行为疗法联合百乐眠胶囊对脑卒中后睡眠障碍患者睡眠质量和血清神经递质水平的影响. 临床医学研究与实践 2021, 6.(combined with cognitive behavioral therapy)

5. 尤峰. 育阴宁神汤对老年脑卒中后睡眠障碍患者睡眠质量及康复效果的影响. 中医临床研究.(combined with psychotherapy)

10) using duplicate data: 1

1. 高振忠; 宋立公. 柴胡龙牡胶囊治疗急性中风后失眠. 中医临床研究 2020, 12, 61.
