# Supplementary material for: Traditional East Asian Herbal Medicine for Post-Stroke Insomnia: A Systematic Review and Meta-Analysis of Randomized Controlled Trials
Source: Int J Environ Res Public Health. 2022 Feb 3;19(3):1754. doi: 10.3390/ijerph19031754 (PMC8834856; doi:10.3390/ijerph19031754)
Supplement: Supplementary file 1 [file ijerph-19-01754-s001.zip › Table S4.pdf]

Table S4. Details of herbal medicine used

Study ID	Name	Dosage form	Main component (per day)	Additional component (per day)
Chen(2010) [39]	Bailemian capsules	capsule	Bulbus Lilii, Radix et Rhizoma seu Caulis Acanthopanacis Senticosi, Radix Flos Albiziae, Concha Margaritifera, Gypsum Fibrosum, Semenae, Foria, Radix, Radix Pheretima, Radix Ophiopogonis, Fructus Schisandrae Chinensis, Medulla Junci, Radix et Rhizoma Salviae Miltiorrhizae	Not applicable
Chen(2018) [40]	Jiawei Buyang Huanwu Decoction	decoction	Radix 120g, Paeoniae Radix rubra 5g, Radix Angelicae Sinensis 6g, Pheretima 3g, Rhizoma Chuanxiong 3g, Semen Persicae 3g, Herba Epimedii 15g, Flos Carthami 3g, Fructus Corni 15g, Caulis Spatholobi 20g, Concha Margaritifera 20g, Rhizoma Acori Tatarinowii 10g, Flos Albiziae 10g, Fructus Gardenia 10g, Semenae 30g, Caulis Plygoni Multiflori 30g	Not applicable
Dai(2020) [41]	Ningxin Anshen Formula	decoction	Semenae 60g, Radix et Rhizoma seu Caulis Acanthopanacis Senticosi 45g, Spica Prunellae 10g	Not applicable
Gao(2016) [42]	Yudian Decoction	decoction	Rhizoma Pinelliae 15g, Rhizoma Arisaematis 15g, Semen Pharbitidis 10g, Rhizoma Spargani 15g, Rhizoma Curcumae 15g, Semen Arecae 15g, Radix et Rhizoma Rhei 10g, Herba Artemisiae Scopariae 15g	Not applicable
Gao(2021) [43]	Two kinds of HM; Diurnal prescription(at 0.5 h after breakfast), Night prescription(at 0. 5 h after dinner)	decoction	Diurnal prescription: Ramulus Cinnamomi 10g, Radix Puerariae Lobatae 10g, Polyporus 15g, Rhizoma Alismatis 15g, Rhizoma Pinelliae 10g, Radix et Rhizoma Salviae Miltiorrhizae 15g, Radix Codonopsis 15g<br>Night prescription: Concha Margaritifera 30g, Magenetitum 10g, Fossilia Ossis Mastodi 20g, Semenae 15g, Hoelen cum Radix 15g, Semen Platycladi 15g, Radix Curcumae 15g, Bulbus Lilii 20g	Not applicable
Guo(2017) [44]	Chaiqin Wendan Decoction	decoction	Radix Bupleuri 15g, Radix Scutellariae 12g, Pericarpium Citri Reticulatae 15g, Fructus Aurantii Immaturus 10g, Caulis Bambusae in Taenia 12g, Semenae 15g, Rhizoma Acori Tatarinowii 15g, Rhizoma Pinelliae 6g, Foria 20g, Radix 12g, Radix et Rhizoma Glycyrrhizae 6g	Not applicable
Huang(2016) [45]	Modified Suanzaoren Decoction	decoction	Zizyphi Spinosi Semen 30g, Radix Rehmanniae Preparata 15g, Fructus Corni 15g, Rhizoma Chuanxiong 20g, Rhizoma Anemarrhenae 10g, Foria 15g, Cortex Albiziae 20g, Caulis Plygoni Multiflori 30g, Radix et Rhizoma Glycyrrhizae 6g	− Dizziness: Radix Angelicae Sinensis, Radix Paeoniae Alba、Fructus Lycii<br>− Dryness of the throat and mouth because of deficiency fire: Radix Ophiopogonis, Radix Rehmanniae for yin-tonifying and clear heat

				− Sleep fright: Borneolum, Concha Margaritifera for settle fright and tranquilize<br>− Night sweating: Fructus Schisandrae Chinensis, Concha Ostreae for sweat-constraining and tranquilizing<br>− liver qi depression: Rhizoma Cyperi, Radix Curcumae
Li(2013) [46]	Zhuyu Anshen Decoction	decoction	Zizyphi Spinosi Semen 30g, Caulis Plygoni Multiflori 30g, Radix Bupleuri 12g, Fructus Aurantii Immaturus 6g, Radix Paeoniae Alba 10g, Semen Persicae 10g, Flos Carthami 10g, Radix Angelicae Sinensis 10g, Radix Rehmanniae 10g, Rhizoma Chuanxiong 6g, Radix Platycodonis 10g, Radix Achyranthis Bidentatae 10g, Rhizoma Corydalis 10g	Not applicable
Liu(2011) [47]	Modified Xuefu Zhuyu Decoction	decoction	Radix Angelicae Sinensis 12g, Rhizoma Chuanxiong 15g, Radix Paeoniae rubra15g, Radix Rehmanniae 15g, Semen Persicae 9g, Flos Carthami 9g, Radix Bupleuri 12g, Radix Achyranthis Bidentatae 15g, Fructus Aurantii Immaturus 15g, Radix Platycodonis 15g, Radix et Rhizoma Glycyrrhizae 9g	− Qi deficiency: Radix Astragali 30g, Radix Codonopsis 15g<br>− Kidney yin deficiency: Fructus Lycii 10g, Fructus Ligustri Lucidi 10g
Mei(2016) [48]	Modified Chaihu Longgumuli Decoction	decoction	Radix Bupleuri 15g, Fossilia Ossis Mastodi 30g, Concha Ostreae 30g, Magenetitum 20g, Radix Scutellariae 10g, Rhizoma Pinelliae 10g, Ramulus Cinnamomi 10g, Radix Codonopsis10g, Foria 10g, Rhizoma Zingiberis 3g, Fructus Jujubae 5	Not applicable
Qin(2014) [49]	Self-made Chinese herbal decoction	decoction	Radix Rehmanniae 20g, Radix Rehmanniae Preparata 20g, Fructus Corni 16g, Fructus Lycii 10g, Fructus Ligustri Lucidi 10g, Rhizoma Chuanxiong 10g, Flos Carthami 10g, Radix Paeoniae Alba 15g, Radix Ophiopogonis 10g, Caulis Plygoni Multiflori 30g, Cortex Albiziae 10g, Semen Zizyphi Spinosae 16g, Radix et Rhizoma Salviae Miltiorrhizae 16g, Hoelen cum Radix 15g	− Qi deficiency: Radix Pseudostellariae 20g, Rhizoma Atractylodis Macrocephalae 16g<br>− Excessive sweating: Fossilia Ossis Mastodi 10g, Concha Ostreae 10g<br>− Deficiency heat: Rhizoma Anemarrhenae 10g, Carapax et Plastrum Testudinis 10g

				− Exuberant heat: Radix Scutellariae 10g, Fructus Gardeniae 10g<br>− Liver qi depression: Radix Bupleuri 10g, Radix Curcumae 10g, Rhizoma Cyperi 10g<br>− Dyspepsia: Fructus Hordei Germinatus 10g, Endothelium Corneum Gigeriae Galli 10g
Qiu(2019) [50]	Changzhi Huanglian Wendan decoction	decoction	Rhizoma Acori Tatarinowii 12g, Radix Radix Polygalae 15g, Rhizoma Coptidis 10g, Caulis Bambusae in Taenia 6g, Aurantii Immaturus 10g, Rhizoma Pinelliae 6g, Pericarpium Citri Reticulatae 12g, Foria 20g, Succinum 3g, Fructus Gardeniae 10g, Fossilia Ossis Mastodi 30g, Concha Ostreae 30g, Concha Margaritifera 30g, Radix et Rhizoma Glycyrrhizae 6g	Not applicable
Su(2013) [51]	Chaihu Longmu pill	pill	Radix Bupleuri, Ginseng Radix, Rhizoma Pinelliae, Cinnamomi Ramulus, Scutellariae Radix, Poria Sclerotium, Concha Ostreae, Rhei Radix et Rhizoma, Zingiberis Rhizoma, Zizyphi Fructus	Not applicable
Wang(2019a) [52]	Huanglian Wendan granule	granule	Rhizoma Coptidis, Rhizoma Pinelliae, Caulis Bambusae in Taenia, Foria, Aurantii Immaturus	Not applicable
Wang(2019b) [53]	Jiawei Jiaotai decoction	decoction	Rhizoma Coptidis 3g, Cinnamomi Cortex Spissus 6g, Radix Scutellariae 6g, Hoelen cum Radix 15g, Rhizoma Atractylodis Macrocephalae 12g, Rhizoma Dioscoreae 20g, Semen Zizyphi Spinosae 15g, Caulis Polygoni Multiflori 15g, Flos Albiziae 6g, Radix et Rhizoma Glycyrrhizae 6g	− Yin deficiency: Radix Glehniae<br>− Inability to sleep: Concha Margaritifera, Fossilia Ossis Mastodi<br>− Liver qi depression: Rhizoma Cyperi, Radix Curcumae<br>− Phlegm-dampness obstructing: Rhizoma Pinelliae, exocarpium Citri Grandis
Xu(2011) [54]	Nourishing kidney yin and promoting	decoction	Radix Rehmanniae 15g, Radix Rehmanniae Preparata 20g, Fructus Corni 15g, Fructus Lycii 15g, Radix Ophiopogonis 15g, Radix Cyathulae 10g,	Not applicable

	blood circulation		Radix et Rhizoma Salviae Miltiorrhizae 15g, Radix Astragali 30g, Radix Angelicae Sinensis 15g, Radix Paeoniae rubra 20g, Radix Paeoniae Alba 20g, Rhizoma Chuanxiong 20g, Caulis Plygoni Multiflori 15g, Fossilia Ossis Mastodi 30g, Concha Ostreae 30g, Rhizoma Acori Tatarinowii 15g, Radix et Rhizoma Glycyrrhizae 15g, Fructus Jujubae 10	
Xu(2012) [55]	Xin Yue Kang pian	pill	Ramulus Uncariae cum Uncis, Semen Zizyphi Spinosae, Rauwolfia serpentina	Not applicable
Xu(2019) [56]	Experience prescription of professor Wang Qiaochu	decoction	Rhizoma Gastrodiae 15g, Radix Bupleuri 15g, Radix Paeoniae rubra 15g, Radix Paeoniae Alba 15g, Radix et Rhizoma Salviae Miltiorrhizae 15g, Semen Persicae 15g, Radix Curcumae 15g, Cortex Albiziae 15g, Rhizoma Acori Tatarinowii 15g, Rhizoma Chuanxiong 12g, Radix Sophorae Flavescentis 12g, Ramulus Uncariae cum Uncis 30g, Radix Puerariae Lobatae 30g, Fossilia Ossis Mastodi 30g, Caulis Plygoni Multiflori 30g, Fructus Tritici Aestivi Levis 30g, Bombyx Batryticatus9g, Flos Carthami 6g, Periostracum Cicadae 6g, Radix et Rhizoma Glycyrrhizae 6g	Not applicable
Yin(2017) [57]	Nourishing kidney and activate blood	decoction	Concha Ostreae 30g, Fossilia Ossis Mastodi 30 g, Radix Rehmanniae Preparata 20g, Radix Paeoniae rubra 20g, Radix Paeoniae Alba20g, Rhizoma Chuanxiong 20g, Radix Rehmanniae 15g, Fructus Corni 15g, Fructus Lycii 15g, Radix Ophiopogonis 15g, Radix et Rhizoma Salviae Miltiorrhizae 15g, Radix Angelicae Sinensis 15g, Rhizoma Acori Tatarinowii 15g, Radix et Rhizoma Glycyrrhizae 15g, Radix Cyathulae 10g, Fructus Jujubae 10g	Not applicable
Zhang(2011) [58]	Guben Jiannao fluid	decoction	Radix Codonopsis 30g, Foria 15g, Fructus Lycii 15g, Fructus Crataegi 12g, Semen Zizyphi Spinosae 15g	Not applicable
Zhang(2017a) [60]	Insomnia granules	granule	Radix Bupleuri 15g, Cortex Moutan 15g, Fructus Gardeniae 10g, Radix Scutellariae 10g, Radix Angelicae Sinensis 12g, Rhizoma Chuanxiong 12g, Radix Paeoniae Alba 15g, Semen Zizyphi Spinosae 15g, Semen Platycladi 15g, Radix Polygalae 10g, Hoelen cum Radix 10g, Rhizoma Atractylodis Macrocephalae 10g, Cortex Albiziae 15g, Radix Curcumae 15g, Radix et Rhizoma Nardostachyos 6g, Fossilia Ossis Mastodi 20g, Concha Ostreae 20g	Not applicable
Zhang(2017b) [59]	Self-made Tongluo Yisui Decoction	decoction	Semen Persicae 10g, Rhizoma Chuanxiong 15g, Radix Angelicae Sinensis 15g, Radix Paeoniae rubra 9g, Radix Rehmanniae 10g, Radix Bupleuri 6g, Radix Puerariae Lobatae 15g, Pheretima 15g, Fructus Aurantii 10g, Radix Achyranthis Bidentatae 10g, Radix Polygalae 5g, Rhizoma Acori Tatarinowii 10g, Fructus Alpiniae Oxyphyllae 10g, Radix	Not applicable

			et Rhizoma Glycyrrhizae 10g	
Zhao(2021) [61]	Modified Chai Shao Long Mu Decoction	Decoction	Radix Bupleuri 12g, Radix Paeoniae Alba 24g, Fossilia Ossis Mastodi 24g, Concha Ostreae 24g, Rhizoma Polygonati Odorati 15g, Foria 12g, Radix et Rhizoma Glycyrrhizae 6g, Semen Zizyphi Spinosae 15g, Radix Angelicae Sinensis 10g, Caulis Plygoni Multiflori 30g	Not applicable
Zhou(2012) [62]	Self-made Yang Xin Fang	decoction	Pheretima 15g, Rhizoma Pinelliae 15g, Caulis Bambusae in Taenia 15g, Hoelen cum Radix 15g, Radix Ophiopogonis 15g, Flos Albiziae 15g, Radix Rehmanniae Preparata 10g, Rhizoma Acori Tatarinowii 10g, Fructus Schisandrae Chinensis 6g	Not applicable
