# Supplementary material for: Traditional East Asian Herbal Medicine for Post-Stroke Insomnia: A Systematic Review and Meta-Analysis of Randomized Controlled Trials
Source: Int J Environ Res Public Health. 2022 Feb 3;19(3):1754. doi: 10.3390/ijerph19031754 (PMC8834856; doi:10.3390/ijerph19031754)
Supplement: Supplementary file 1 [file ijerph-19-01754-s001.zip › Table S5.pdf]

Table S5. Frequencies of usage of single herb in HM prescriptions

Name of single herb	frequency
Zizyphi Spinosae Semen	11
Glycyrrhizae Radix	9
Rhizoma Salviae Miltiorrhizae	9
Poria(Hoelen)	8
Acori Graminei Rhizoma	8
Ligustici Rhizoma	8
Bupleuri Radix	7
Polygoni Multiflori Ramuls	7
Paeoniae Radix Alba	6
Bovis Ossis Fossilia	6
Paeoniae Radix Rubra	6
Aurantii Immaturus Fructus	6
Persicae Semen	5
Ophiopogonis Radix	5
Ostreae Concha	5
Pinelliae Rhizoma	5
Corni Fructus	5
Rehmanniae Radix Siccus	5
Rehmanniae Radix Preparat	5
Achyranthis Radix	5
Lycii Fructus	4
Polygalae Radix	4
Bambusae Caulis In Taeniam	4
Cristariae Concha	4
Flos Albiziae	4
Carthami Flo	4
Puerariae Radi	3
Angelicae Gigantis Radix	3
Codonopsis Pilosulae Radix	3
Jujubae Fructus	3
Allolobophora Corpus	3
Albizziae Cortex	3

Note. We included single herb used more than 3 times in the Table.
